# Supplementary figures and images for: CRISPRi-based screen of autism spectrum disorder risk genes in microglia uncovers roles of ADNP in microglia endocytosis and synaptic pruning
Source: Mol Psychiatry. 2025 Apr 6;30(9):4176–93. doi: 10.1038/s41380-025-02997-z (PMC12339388; doi:10.1038/s41380-025-02997-z)

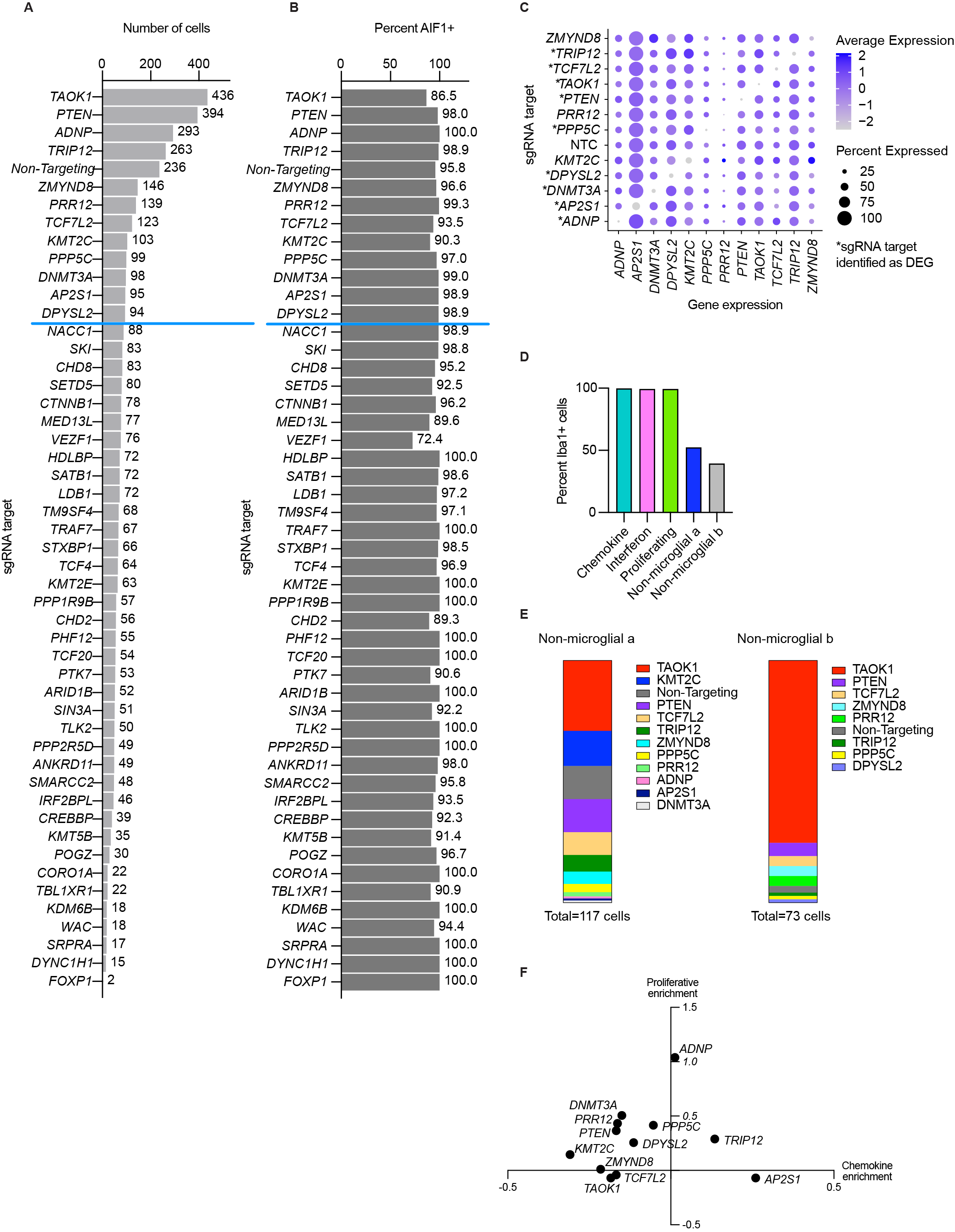

Supplement: Supplementary file 1 — Supplemental Figure 1 [file 41380_2025_2997_MOESM1_ESM.png]

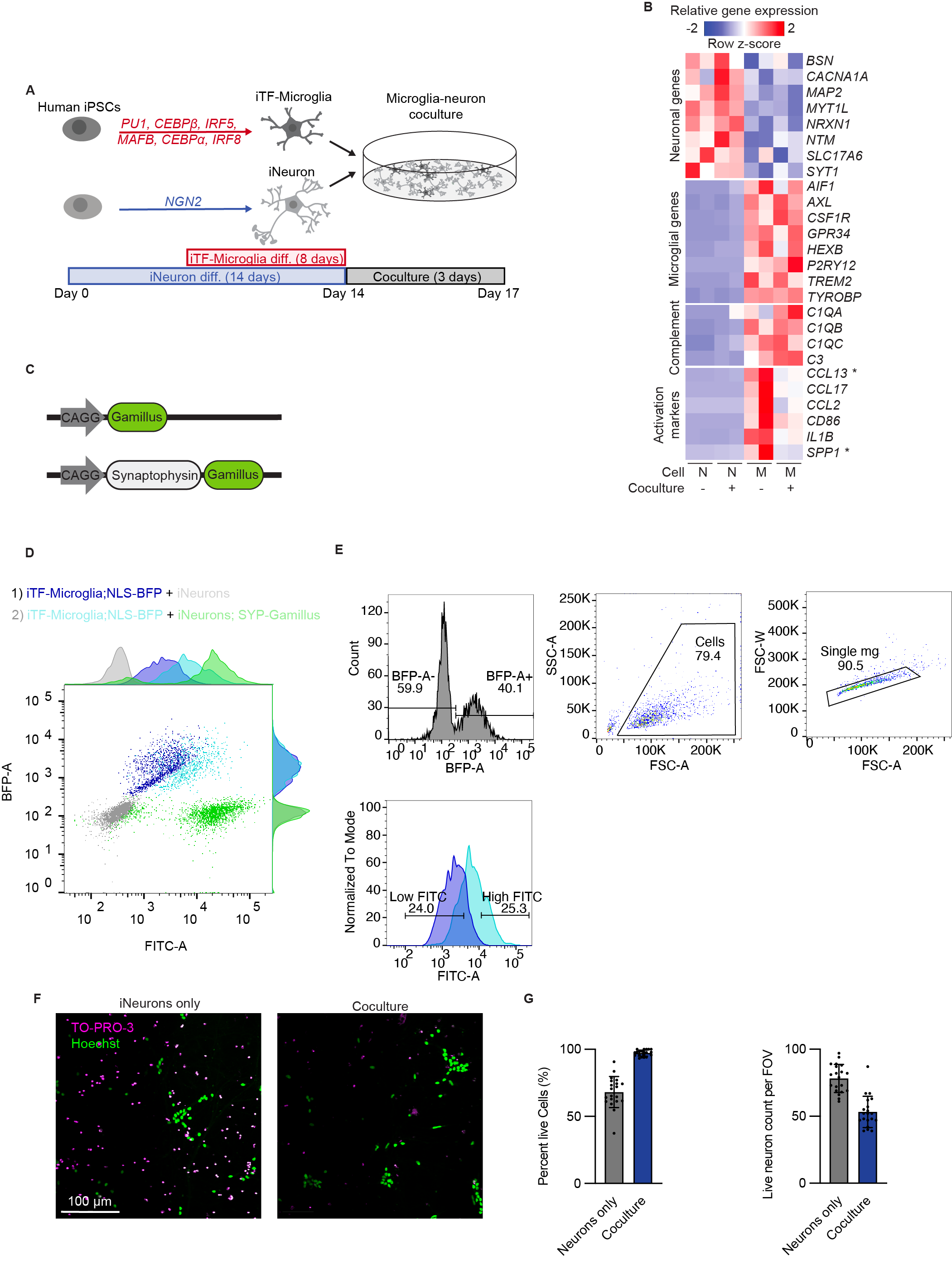

Supplement: Supplementary file 2 — Supplemental Figure 2 [file 41380_2025_2997_MOESM2_ESM.png]

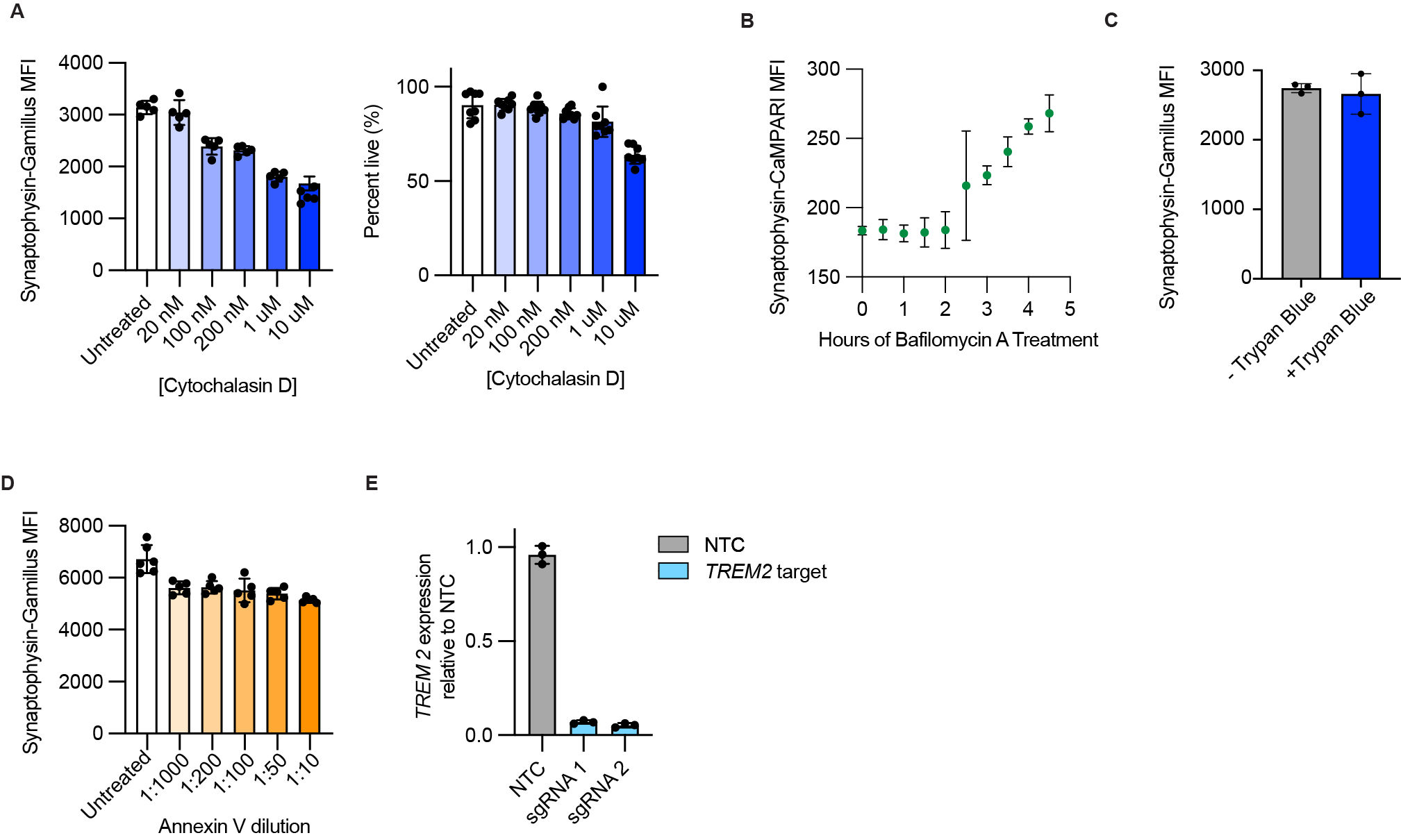

Supplement: Supplementary file 3 — Supplemental Figure 3 [file 41380_2025_2997_MOESM3_ESM.png]

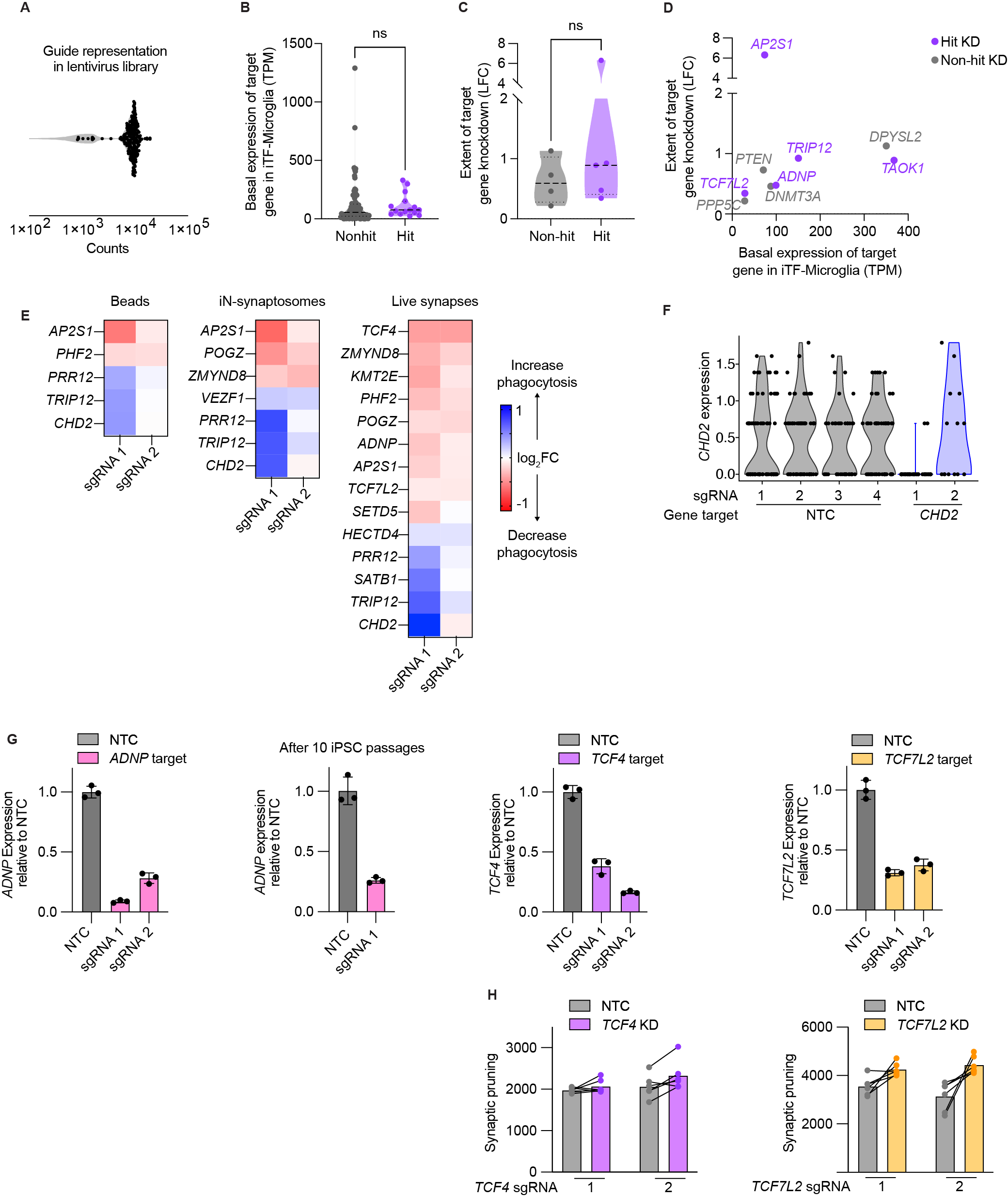

Supplement: Supplementary file 4 — Supplemental Figure 4 [file 41380_2025_2997_MOESM4_ESM.png]

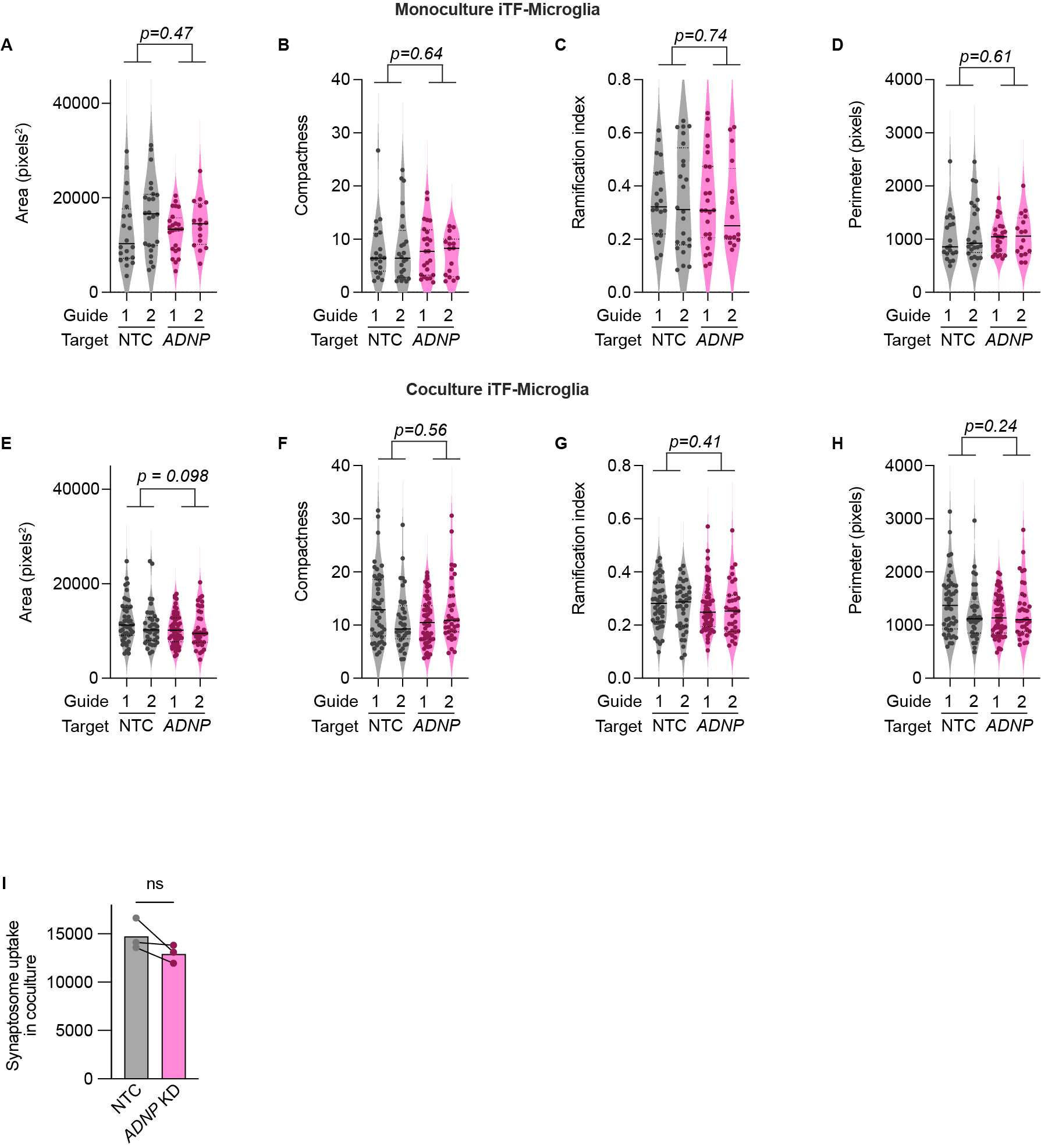

Supplement: Supplementary file 5 — Supplemental Figure 5 [file 41380_2025_2997_MOESM5_ESM.png]

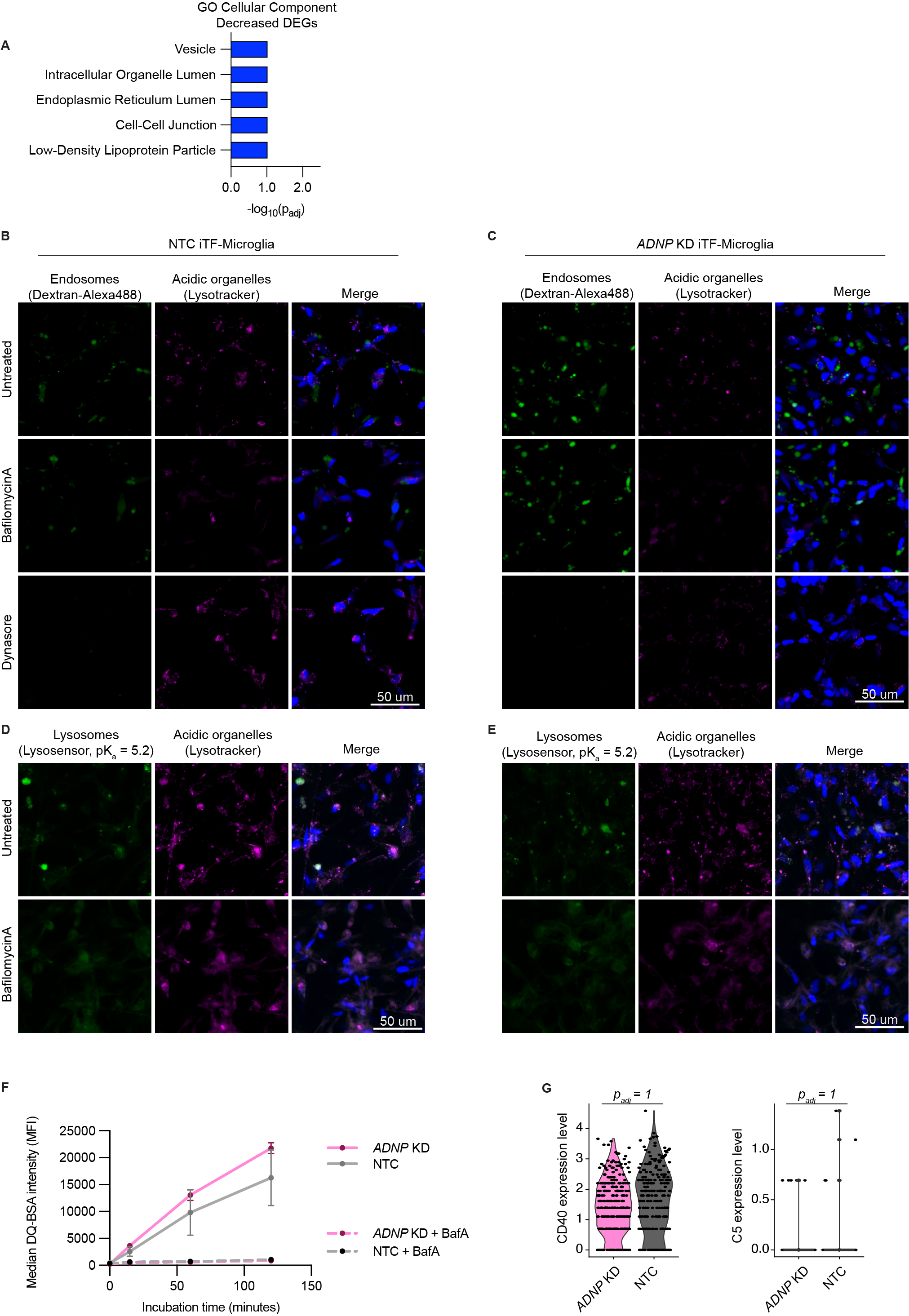

Supplement: Supplementary file 6 — Supplemental Figure 6 [file 41380_2025_2997_MOESM6_ESM.png]

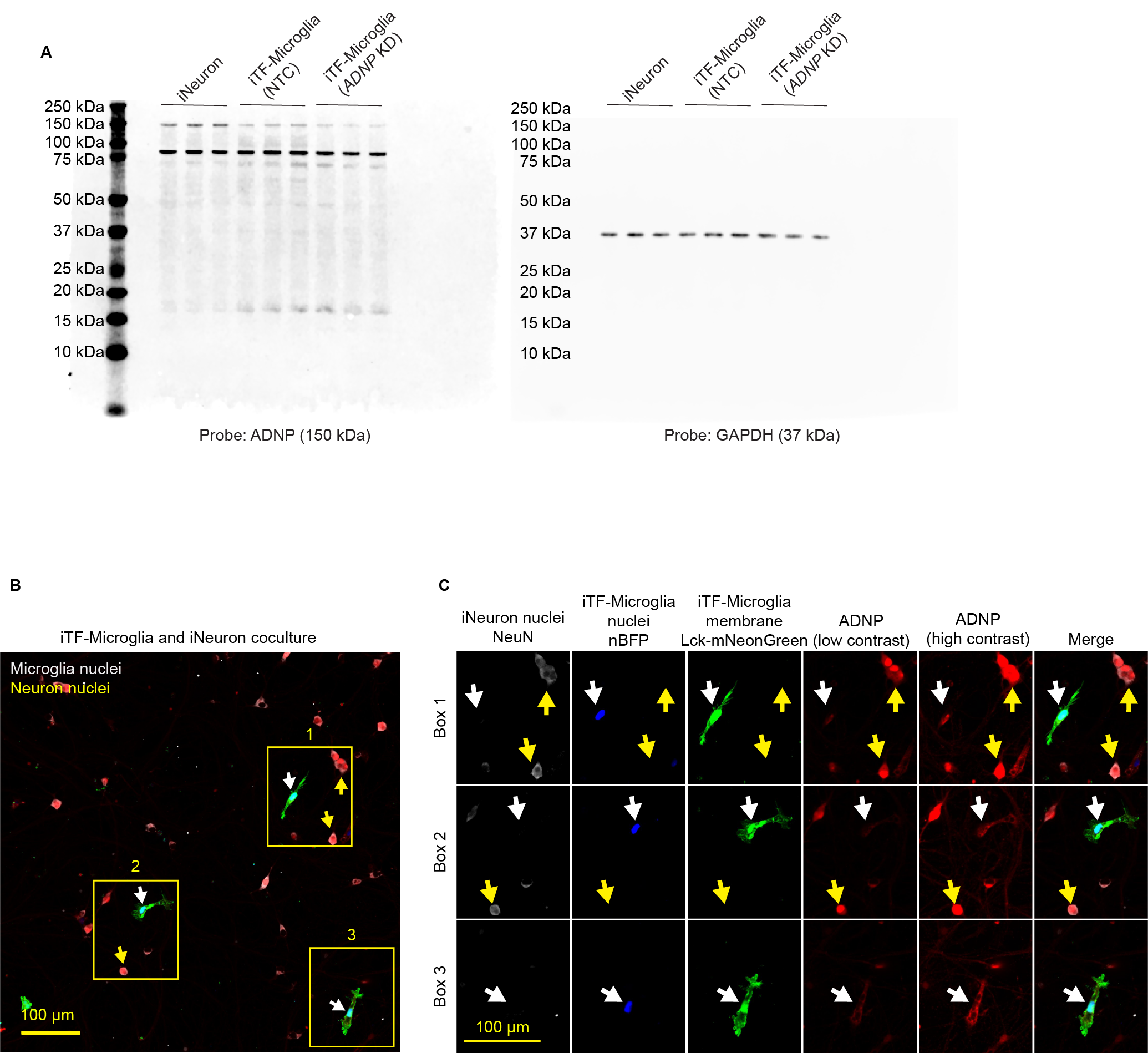

Supplement: Supplementary file 7 — Supplemental Figure 7 [file 41380_2025_2997_MOESM7_ESM.png]
